# Intraoperative imaging of hepatic cancers using γ-glutamyltranspeptidase-specific fluorophore enabling real-time identification and estimation of recurrence

**DOI:** 10.1038/s41598-017-03760-3

**Published:** 2017-06-14

**Authors:** Yoichi Miyata, Takeaki Ishizawa, Mako Kamiya, Suguru Yamashita, Kiyoshi Hasegawa, Aya Ushiku, Junji Shibahara, Masashi Fukayama, Yasuteru Urano, Norihiro Kokudo

**Affiliations:** 1 Hepato-Biliary-Pancreatic Surgery Division, Department of Surgery, Graduate School of Medicine, The University of Tokyo, Tokyo, Japan; 2 Department of Gastroenterological Surgery, Cancer Institute Hospital, Japanese Foundation for Cancer Research, Tokyo, Japan; 3 Laboratory of Chemical Biology and Molecular Imaging, Graduate School of Medicine, The University of Tokyo, Tokyo, Japan; 4 PRESTO, Japan Science and Technology Agency, Saitama, Japan; 5 Department of Pathology, Graduate School of Medicine, The University of Tokyo, Tokyo, Japan; 6 Graduate School of Pharmaceutical Sciences, The University of Tokyo, Tokyo, Japan; 7 CREST, Japan Agency for Medical Research and Development, Tokyo, Japan

## Abstract

γ-Glutamyltranspeptidase (GGT) is upregulated in a variety of human cancers including primary and secondary hepatic tumors. This motivated us to use γ-glutamyl hydroxymethyl rhodamine green (gGlu-HMRG), a novel fluorophore emitting light at around 520 nm following enzymatic reaction with GGT, as a tool for the intraoperative identification of hepatic tumors. gGlu-HMRG was topically applied to 103 freshly resected hepatic specimens. Fluorescence imaging using gGlu-HMRG identified hepatic tumors with the sensitivity/specificity of 48%/96% for hepatocellular carcinoma, 100%/100% for intrahepatic cholangiocarcinoma, and 87%/100% for colorectal liver metastasis. High gGlu-HMRG fluorescence intensity was positively associated with the incidence of microscopic vascular invasion in HCC and the risk of early postoperative recurrence in CRLM. These results suggest that gGlu-HMRG imaging could not only be a useful intraoperative navigation tool but also provide information related to postoperative disease recurrence.

## Introduction

There is an urgent need for reliable modalities that enable accurate intraoperative identification of cancer tissues to both enhance the resection rate and improve postoperative survival outcomes. Among the image guidance tools currently in development, *in vivo* fluorescence imaging has the potential to assist surgeons in identifying anatomical structures during surgery. However, only a few agents, such as indocyanine green (ICG) and 5-aminolevulinic acid, have advanced to clinical use^1, 2^.

In the case of hepatic cancers, ICG can be used as a fluorescence probe for intraoperative identification of hepatic tumors, because preoperatively injected ICG accumulates in the cancerous tissues of hepatocellular carcinoma (HCC) and in the non-cancerous hepatic parenchyma around metastatic tumors, where biliary excretion disorders exist^1, 3^. ICG fluorescence imaging starts being used in clinical settings widely especially during laparoscopic liver resection, where intraoperative tumor detection by visual inspection and palpation of the hepatic surfaces is limited compared to open surgery. In contrast, ICG fluorescence imaging of hepatic cancers has limitations in insufficient specificity (around 40% in cirrhotic patients), because this technique is based on cancer-nonspecific biliary excretion disorders and unable to visualize hepatic adenocarcinoma tissues directly.

γ-Glutamyltranspeptidase (GGT; EC 2.3.2.2) is a cell-surface enzyme involved in glutathione homeostasis in virtually all cells^4^. Interestingly, GGT is frequently upregulated in a variety of human cancer tissues^5, 6^, and its role in ensuring an adequate supply of cysteine for intracellular glutathione synthesis^7^ is thought to promote rapid tumor growth and acquisition of drug resistance^8–10^. In order to visualize GGT activity in living samples, several GGT-activatable fluorescent probes have been developed^x,y^. Among these probes, we focused on our previously developed γ-glutamyl hydroxymethyl rhodamine green (gGlu-HMRG), which enables *in vivo* identification of cancer tissues^11, 12^. gGlu-HMRG is nonfluorescent in its native state but is rapidly hydrolyzed by GGT to generate HMRG, which emits fluorescence with a peak wavelength of ~520 nm^12^. So far, we have demonstrated efficacy of cancer imaging with topical administration of gGlu-HMRG using various human cancer tissues such as breast cancer^13, 14^, head and neck cancer^15^, lung cancer^16^, primary colorectal cancer^17^, pancreatic cancer^18^ and oral cancer^19^. Because overexpression of GGT has been observed in HCC and intrahepatic cholangiocarcinoma (ICC)^6, 20–22^, we hypothesized that gGlu-HMRG could also be used for identification of hepatic malignancies during liver resection. In this report, we indicate that fluorescence imaging following topical administration of gGlu-HMRG enables rapid and highly specific identification of human primary and secondary hepatic cancers in fresh resected specimens. In addition, the present study also suggests that fluorescence imaging using gGlu-HMRG may provide information on the biological characteristics of cancerous tissues that is associated with a higher risk postoperative disease recurrence.

## Results

### Patient clinicopathological characteristics

The preoperative characteristics of the 95 patients enrolled in our study are summarized in Supplementary Table S1. Of these, 48 patients were diagnosed with HCC and 47 patients with adenocarcinoma (8 patients with ICC and 39 patients with colorectal cancer with liver metastasis [CRLM]). The HCC group included more men (85% vs. 60%, *P* = 0.005), patients with hepatitis B or C infection (75% vs. 13%, *P* < 0.001), pathologically diagnosed liver cirrhosis (LC) (40% vs. 0%, *P* < 0.001), and unfavorable ICG retention rate at 15 min (ICGR15) values (median [range] 11.8% [5.20–41.2%] vs. 8.1% [4.00–21.7%], *P* < 0.001; normal <10%).

### Fluorescence intensities of tissue samples after administration of gGlu-HMRG

**Figure 1:**
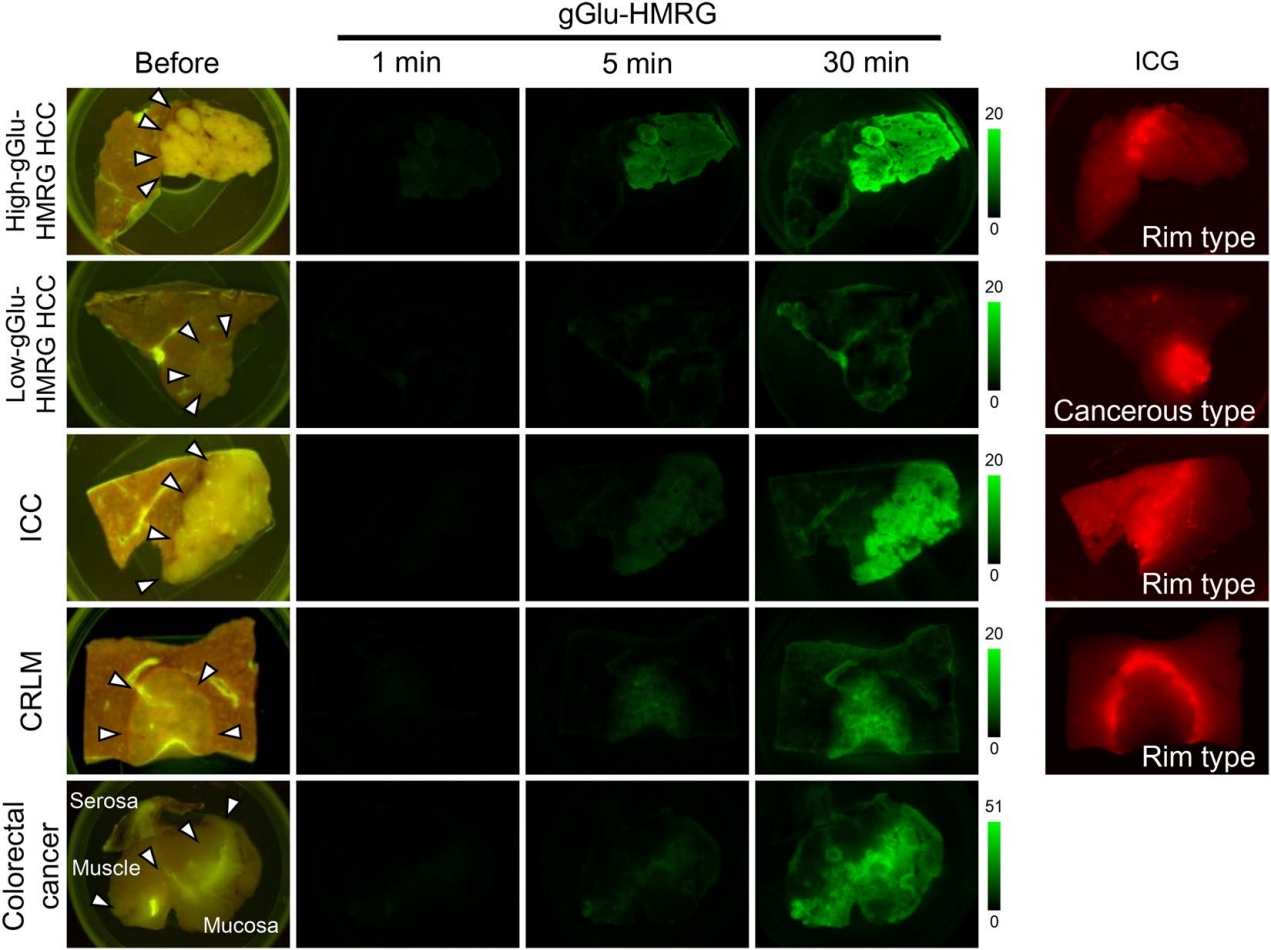
Fluorescence imaging of HCC, ICC, and CRLM using gGlu-HMRG. Fluorescence images of representative resected tumor samples acquired before and at 1, 5, and 30 min after spraying with gGlu-HMRG. Images were obtained with the blue filter setting (excitation 445–490 nm, emission 515 nm, long pass). The High-gGlu-HMRG HCC, ICC, and CRLM were identified from 5 min after topical administration of gGlu-HMRG, while Low-gGlu-HMRG HCC remained unidentifiable even at 30 min after the imaging. Arrows indicate boundaries of the tumors by macroscopic gross examination. Right-most column shows fluorescence images of the same tumor samples acquired by near-infrared imaging (excitation 710–750 nm, emission 810 nm, long pass). “Cancerous type” indicates a fluorescence signal from the tumor and “rim type” indicates a fluorescence signal from the non-cancerous hepatic parenchyma but not the tumor itself.

**Figure 2:**
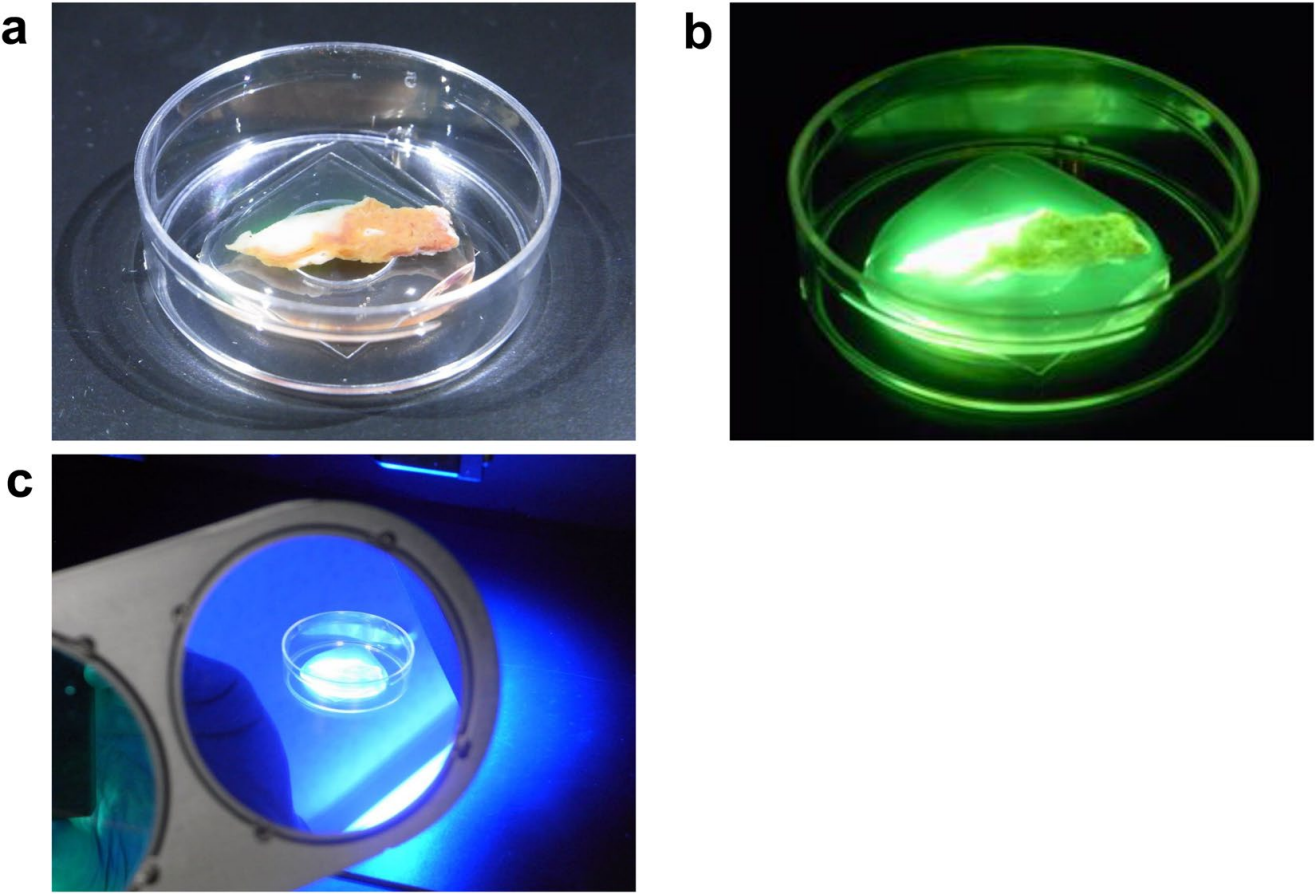
Naked-eye observation of fluorescence signals following topical administration of gGlu-HMRG on resected hepatic tissues. (**a**) gGlu-HMRG was sprayed on to cut surface of a resected hepatic specimen including ICC. (**b**) Cancerous tissues of ICC were identified as a highly fluorescing legion by naked-eye examination. (**c**) Fluorescence signals were observed through a filter glass (515 nm, long pass) under blue-light illumination (445–490 nm).

A total of 103 hepatic tumors (HCC *n* = 50, ICC *n* = 8, CRLM *n* = 45) and 10 primary colorectal cancer tumors (from 9 of the CRLM patients) were resected and subjected to fluorescence imaging. For fluorescence imaging of gGlu-HMRG, fresh tumor samples were sprayed with a solution of gGlu-HMRG (within 10 min of removal), and imaged at 1, 5, and 30 min after spraying (Fig. 1). Fluorescence signals visualized on a monitor of the *in vivo* fluorescence imaging system were also identifiable by naked-eye examinations through a light-blocking glasses (emission 515 nm, long pass, Fig. 2).

**Figure 3:**
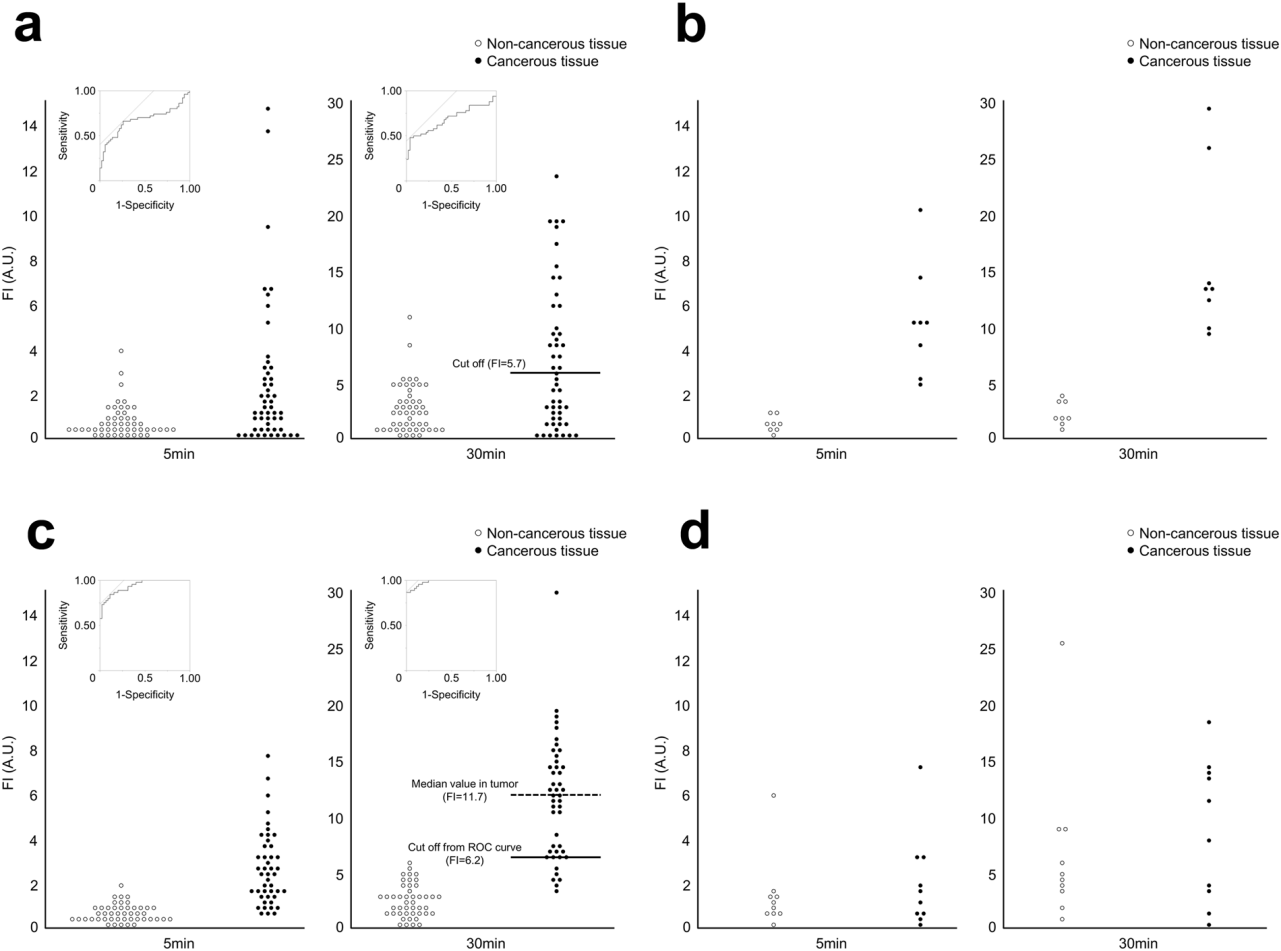
Sensitivity and specificity of gGlu-HMRG fluorescence imaging for detection of cancerous tissues. Scatter plots of the fluorescence intensities (FIs, in AU) of cancerous tissue and non-cancerous background tissues measured 5 and 30 min after spraying of HCC (**a**, *n* = 50), ICC (**b**, *n* = 45), CRLM (**c**, *n* = 8), and primary colorectal cancer (d, n = 10) with gGlu-HMRG. ROC curves were also indicated in HCC (**a**) and CRLM (**c**). The median FIs of primary colorectal cancer and non-cancerous colorectal tissues at 30 min were 9.6 A.U. and 4.5 A.U., respectively (*P* = 0.496, **d**).

Figure 3 shows the distribution of fluorescence intensities (FIs) in the cancerous tissue and non-cancerous liver parenchyma of the HCC, ICC, and CRLM samples. Receiver operating characteristic (ROC) curves were constructed to determine the sensitivity and specificity of FIs at 30 min after gGlu-HMRG administration for diagnosis of HCC, ICC, and CRLM. Based on the calculated ROC cut-off values, the sensitivity/specificity was 48%/96% for HCC (cut-off FI: 5.7 arbitrary units [A.U.]), 100%/100% for ICC (9.0 A.U.), and 87%/100% for CRLM (6.2 A.U.). In the following analysis, HCCs were segregated into High-gGlu-HMRG tumors (n = 24) and Low-gGlu-HMRG tumors (n = 26), based on the cut-off of 5.7. The median (range) FIs of primary colorectal cancer and non-cancerous colorectal tissues at 30 min were 9.6 (0.26–18.2) A.U. and 4.5 (0.90–25.3) A.U., respectively (*P* = 0.496).

### Fluorescence imaging of hepatic tumors using ICG

We also performed near-infrared fluorescence imaging of ICG in the same resected tumors (Fig. 1), using the ICG that had been intravenously administrated for the preoperative liver function test as a fluorescence source. Among the 41 HCC samples examined, 25 tumors showed “cancerous-type” fluorescence (partial or complete fluorescence in the tumors), while the remaining 16 tumors showed “rim-type” fluorescence (fluorescence in the surrounding non-cancerous hepatic tissue but not in the tumors themselves)^1, 2^. Proportion of High-gGlu-HMRG tumors was 48% in cancerous-type HCC and 69% in rim-type HCC (P = 0.192). In contrast, all of the ICC and CRLM tumors showed the rim-type ICG fluorescence signal.

**Figure 4:**
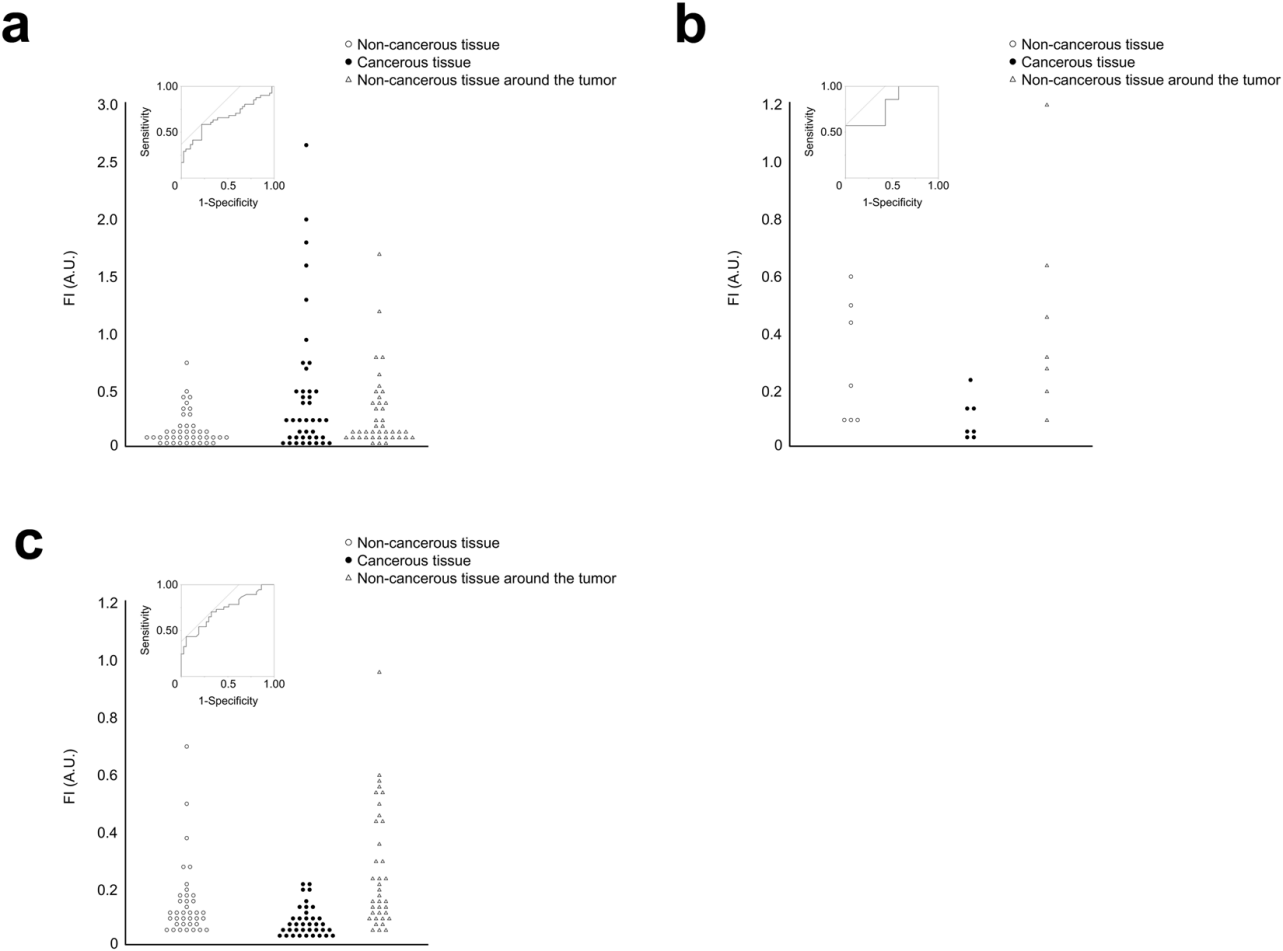
Sensitivity and specificity of ICG fluorescence imaging for detection of hepatic tumors. Scatter plots of the fluorescence intensities (FIs, in AU) of cancerous tissue (**a**, HCC; **b**, ICC; **c**, CRLM), non-cancerous tissue around the tumor, and non-cancerous tissue >5 mm apart from the tumor border in near-infrared imaging following preoperative intravenous injection of ICG. ROC curves in detection of HCC as a fluorescing lesion and ICC and CRLM as a non-fluorescing lesion were also indicated.

FIs in the cancerous tissues were significantly higher in HCC showing “cancerous-type” fluorescence than those in “rim-type” HCC (median FI, 0.40 vs. 0.07, *P* = 0.002). When ROC curves were constructed based on FIs of cancerous tissues and non-cancerous hepatic tissues, the sensitivity/specificity of ICG-fluorescence imaging in detection of HCC was 59%/78% for (cut-off FI: 0.20 A.U.). In contrast, FIs in the cancerous tissues were lower than those of non-cancerous hepatic tissues in all the ICC and CRLM. The sensitivity/specificity of ICG-fluorescence imaging to negatively identify ICC and CRLM was 57%/100% (cut-off IF: 0.05 A.U.) and 70%/68% (0.08 A.U.), respectively (Fig. 4).

### Fluorescence intensities of tissue samples treated simultaneously with gGlu-HMRG and a GGT inhibitor

To confirm that the gGlu-HMRG-derived fluorescence signal was dependent on GGT activity, we imaged tissues after application of a mixture of gGlu-HMRG and GGsTop, a specific and irreversible GGT inhibitor. After addition of the mixture, the fluorescence signals in the cancerous tissues of the HCC, ICC, and CRLM samples were virtually undetectable, whereas they could be clearly identified in paired samples imaged after application of gGlu-HMRG alone (see Supplementary Fig. S2). This was confirmed by quantification of the signals, which showed that FIs at 30 min after administration of gGlu-HMRG with GGsTop were significantly lower than those obtained using gGlu-HMRG alone in all of the hepatic tumors and in the background liver tissues (see Supplementary Fig. S2).

### Immunohistochemical staining of GGT in tumor samples

**Figure 5:**
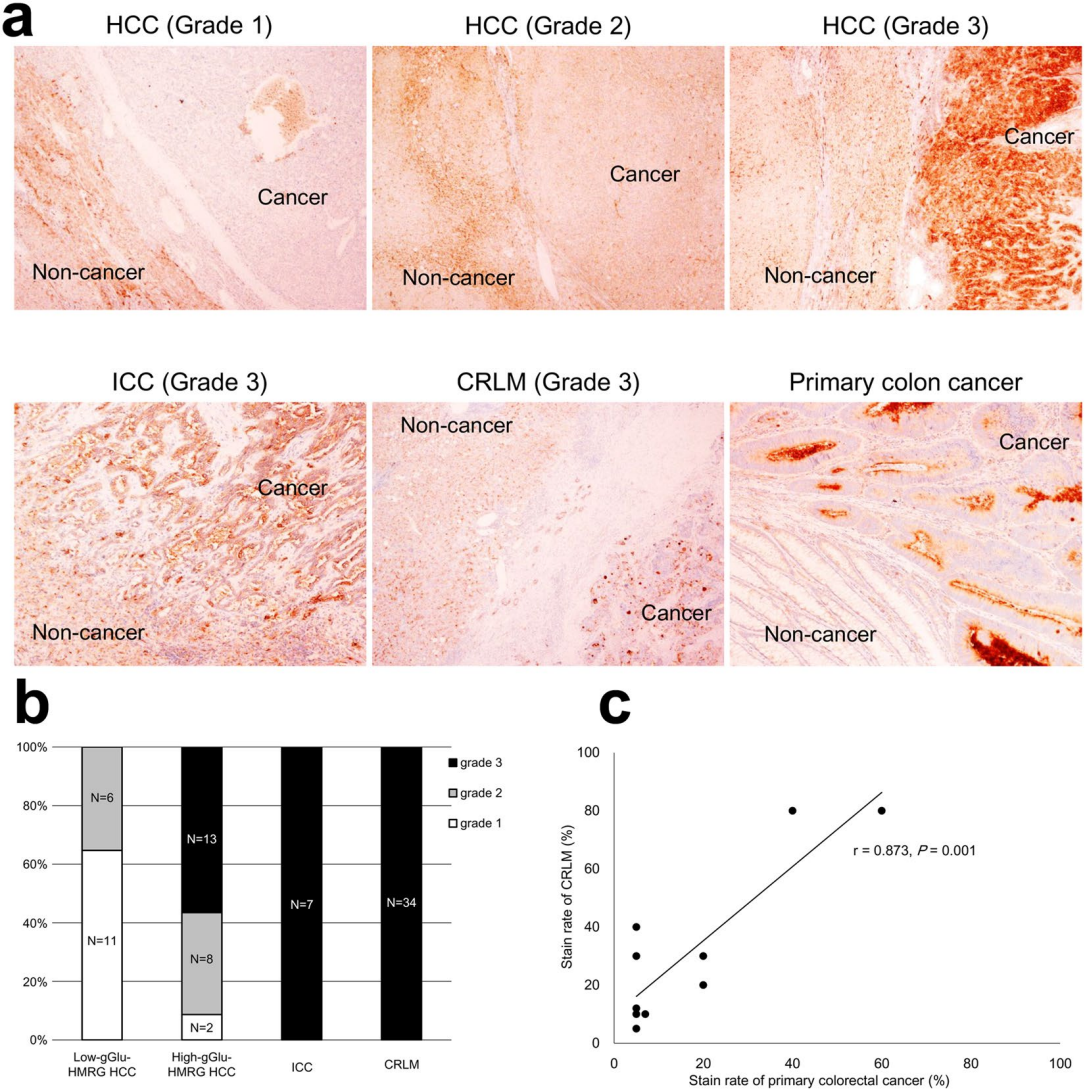
GGT expression in resected tissues. (**a**) Immunohistochemical staining of GGT in tissue samples showing the grades assigned according to the cell membrane staining pattern: Grades 1, 2, and 3 correspond to lower, equivalent, or higher, respectively, staining intensity in cancerous tissues compared with non-cancerous liver parenchyma. (**b**) Proportion of the indicated tumor samples with GGT staining Grades 1, 2, and 3. Staining intensity tended to be higher in 23 High-gGlu-HMRG HCCs (21 samples Grade 2 or 3) than in 17 Low-gGlu-HMRG HCCs (6 samples Grade 2 or 3, *P* < 0.001). All 7 ICCs and 34 CRLMs examined were classified as Grade 3. (**c**) In 9 patients (10 metastatic tumors) with synchronous CRLM, the “stain rate” (proportion of cancerous tissue stained vs. unstained) in the hepatic metastases were positively correlated with and tended to be higher than the stain rates of the corresponding primary colorectal cancers (Pearson’s correlation coefficient *r* = 0.873, *y* = 1.28× + 9.76, *P* = 0.001).

We next examined GGT expression in the resected tumor samples by immunohistochemical staining. GGT staining was scored as one of three grades. Cell-surface staining intensity that was lower, equivalent, or higher in the cancerous areas of the tissues than in the non-cancerous liver parenchyma of the same samples was classified as Grade 1, 2, or 3, respectively (Fig. 5a). We found that the GGT grades tended to be higher for High-gGlu-HMRG HCC (91% of samples Grade 2 or 3) than for Low-gGlu-HMRG HCC (35% of samples Grade 2 or 3, *P* < 0.001; Fig. 5b). Because the GGT staining intensity of all ICC and CRLM tissues was classified as Grade 3, we compared the relative GGT staining by calculating the ratio of the stained to unstained area in the cancerous tissues (“staining rate”). The median staining rates for ICC and CRLM samples were 90% (range 5–90%) and 40% (5–90%), respectively. Interestingly, the staining rates in CRLM samples correlated positively with the rates in the corresponding primary colorectal lesions for all 9 patients (10 metastatic nodules) examined (Pearson’s correlation coefficient *r* = 0.873, *P* = 0.001; Fig. 5c).

### Oncological outcomes of patients stratified according to fluorescence imaging using gGlu-HMRG

Table 1 summarizes the associations between gGlu-HMRG fluorescence imaging of HCC and CRLM samples and tumor-related factors and postoperative outcomes. High-gGlu-HMRG HCCs were associated with higher preoperative α-fetoprotein levels (24.0 ng/mL vs. 6.5 ng/mL, *P* = 0.021) and higher incidence of microscopic vascular invasions (58% vs. 27%, *P* = 0.025). To evaluate the association between CRLM FIs and oncological outcomes, CRLM tumors were segregated into High-gGlu-HMRG (*n* = 23) and Low-gGlu-HMRG (*n* = 22) groups according to the median FI of the cancerous tissues at 30 min after gGlu-HMRG administration (Fig. 3; 11.7 A.U.). We found that High-gGlu-HMRG CRLMs were associated with higher preoperative serum GGT levels (64 U/L vs. 29 U/L, *P* = 0.025) and a higher incidence of early (<6 months) postoperative disease recurrence (45% vs. 16%, *P* = 0.048) (Table 1).

**Table 1:** Association between gGlu-HMRG fluorescence imaging and tumor-related factors and postoperative outcome.

	HCC			CRLM		
	Low-gGlu-HMRG HCC (26 tumors)	High-gGlu-HMRG HCC (24 tumors)	*P* value	Low-gGlu-HMRG CRLM (22 tumors)	High-gGlu-HM	*P* value
					RG CRLM (23 tumors)	
Preoperative blood examinations						
GGT (U/L)	44 (16–317)^a^	50 (16–593)	0.327	29 (13–436)	64 (16–289)	0.025
ICGR15 (%)	12.6 (5.6–41.2)	11.0 (5.2–34.8)	0.328	8.1 (4.0–20.7)	7.95 (4.9–18.9)	0.922
Tumor markers						
AFP (ng/mL)	6.5 (1.9–4652.5)	24.0 (3.0–139921.0)	0.021	—	—	—
DCP (mAU/mL)	65 (9–27193)	224 (8–64225)	0.688	—	—	—
CEA (ng/mL)	3.9 (1.9–26.3)	3.2 (1.0–8.2)	0.381	14.3 (2.5–1087.8)	24.8 (3.8–441.3)	0.448
CA19-9 (U/mL)	18 (1–128)	18 (1–74)	0.991	16 (1–4825)	39 (1–638)	0.422
Pathological findings						
Tumor size (mm)	25 (8–80)	31 (9–98)	0.236	23 (8–77)	26 (8–160)	0.524
Tumor differentiation (well)	11 (42%)	6 (25%)	0.197	6 (27%)	7 (32%)	0.741
Vascular invasion (present)	7 (27%)	14 (58%)	0.025	—	—	—
LN metastasis (present)	—	—	—	11 (58%)	13 (65%)	0.415
UICC TNM stage (III and IV)	1 (4%)	3 (13%)	0.296	17 (89%)^b^	17 (85%)^b^	0.676
KRAS mutation (present)	—	—	—	10 (53%)	4 (20%)	0.188
Recurrence						
Within 6 months of surgery, *n* (%) of patients	2 (8%)	5 (21%)	0.220	3 (16%)	9 (45%)	0.048

^a^Data are the median (range) or *n* (%) of the indicated number of tumors. ^b^UICC 7th edition staging of primary colon cancer. HCC, hepatocellular carcinoma; CRLM, colorectal cancer liver metastasis; GGT, γ-glutamyltranspeptidase; ICGR15, indocyanine green retention test at 15 min; AFP, α-fetoprotein; DCP, des-gamma carboxy prothrombin; CEA, carcinoembryonic antigen; CA19-9, carbohydrate antigen 19-9; LN, lymph node; UICC, Union of International Cancer Control.

**Figure 6:**
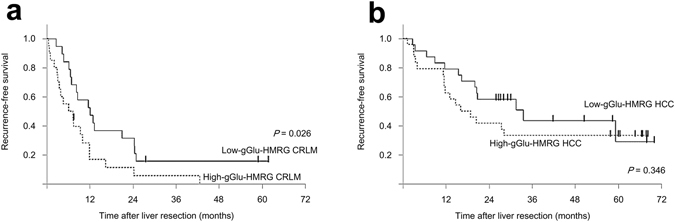
Postoperative recurrence-free survival of CRLM and HCC patients stratified by tumor fluorescence intensity after gGlu-HMRG application. (**a**) Kaplan–Meier curves of recurrence-free survival of patients with tumors classified as Low-gGlu-HMRG CRLMs (*n* = 19, solid line) or High-gGlu-HMRG CRLMs (*n* = 20, dotted line). Two-year recurrence-free survival rates were 32% and 11%, respectively (*P* = 0.026, log-rank test). (**b**) Kaplan–Meier curves of recurrence-free survival of patients with tumors classified as Low-gGlu-HMRG HCCs (*n* = 24, solid line) or High-gGlu-HMRG HCCs (*n* = 24, dotted line). Two-year recurrence-free survival rates were 58% and 42%, respectively (*P* = 0.346, log-rank test).

Recurrence-free survival curves for patients after resection of HCC or CRLM are shown in Fig. 6. For the CRLM group, the recurrence-free survival rate for the 20 patients with tumors classified as High-gGlu-HMRG was significantly lower than for the 19 patients with Low-gGlu-HMRG CRLM tumors (11% vs. 32% at 2 years, *P* = 0.026). In contrast, there was no significant difference in the postoperative recurrence rate of patients with High- or Low-gGlu-HMRG HCC (*n* = 24 both groups). The median follow-up period for HCC and CRLM patients was 57 months and 32 months, respectively.

## Discussion

In this study, we showed that fluorescence imaging using topical administration of gGlu-HMRG on freshly resected specimens enabled rapid identification of hepatic malignancies by gross examination, with 60% detection of HCC and 100% detection of adenocarcinoma. The major advantage of gGlu-HMRG as an intraoperative cancer tracer lies in its rapid response due to the high enzymatic activity of GGT and the regulation of fluorescence at a single enzyme reactive site. Indeed, fluorescence signal became visible 5 min after topical administration of gGlu-HMRG to the hepatic specimen. Another favorable property of gGlu-HMRG imaging is the high tumor-to-background fluorescence ratio. gGlu-HMRG is constitutively nonfluorescent and emits fluorescence only after activation by GGT^23, 24^. Therefore, washout of the probe to reduce background fluorescence is not required for cancer detection. In addition, the imaging wavelength of HMRG lies in the visible range, enabling surgeons and pathologists to perform real-time and direct evaluation of surgical specimens without the need for display monitors.

The ability to visualize hepatic tumors by fluorescence imaging using gGlu-HMRG develops under the upregulated expression of GGT in cancer cell membranes^5^. In this study, the fluorescence signal in cancerous tissues was markedly decreased when a specific irreversible GGT inhibitor was co-administered with gGlu-HMRG. Furthermore, we found that among HCCs, the most intense GGT immunohistochemical staining was observed in samples with higher FIs after administration of gGlu-HMRG. Overall, ICCs showed the highest gGlu-HMRG FIs and very high GGT staining rates, probably because ICC is derived from cholangiocytes, which overexpress GGT. Although GGT staining intensity in CRLM samples was higher in the cancerous tissues than in the background hepatic tissues, the GGT staining rate ranged from 5% to 90% (median 40%). Among the samples of primary colorectal lesions and synchronous CRLMs, the GGT staining rate in CRLM was positively correlated with and tended to be higher than that of the primary colorectal cancers, as suggested by a previous study^25^. Further work will be necessary to clarify whether GGT overexpression in primary colorectal cancer is associated with a high potential for liver metastasis or whether GGT expression levels increase in the CRLMs in the hepatic microenvironment.

Liver resection is a rare surgical procedure where *in vivo* fluorescence imaging of cancer has been applied to clinical settings. In this technique, hepatic cancers are identified from the hepatic surfaces because the ICG that was intravenously injected prior to surgery accumulates in cancerous tissues of differentiated HCC and in non-cancerous hepatic parenchyma around poorly differentiated HCC and adenocarcinoma in the liver, where biliary excretion disorders exist. This cancer-nonspecific imaging based on biliary congestion enables sensitive identification of hepatic cancers, but can lead to an unfavorable false-positive rate (~40% for HCC^17^) and specificity (78% for HCC and 68% for CRLM in the present study). In contrast, the direct reaction between cancer cells and the gGlu-HMRG fluorescent probe easily allowed positive identification of hepatic cancers with specificities of close to 100% for HCC, ICC, and CRLM. In addition, approximately 70% of rim-type HCCs in ICG-fluorescence imaging were highly fluorescing in gGlu-HMRG imaging (High-gGlu-HCC). Thus, the concomitant use of gGlu-HMRG- and ICG-based imaging will enhance both the specificity and sensitivity of fluorescence imaging of tumors during hepatectomy. Fluorescence imaging using gGlu-HMRG may also be useful for intraoperative diagnosis of lymph node metastases and peritoneal dissemination of ICC and colorectal cancer, enabling surgeons to decide on surgical indications before obtaining the results of intraoperative rapid pathological examinations.

Previous studies have revealed that high levels of serum and/or tissue GGT are associated with unfavorable prognosis in cancer patients^26–30^, probably because upregulation of GGT ensures a steady supply of cysteine for glutathione synthesis in cancer cells, leading to rapid tumor growth and acquisition of drug resistance^5, 7^. Indeed, our study revealed higher recurrence rate in High-gGlu-HMRG CRLM which was higher serum GGT levels. And High-gGlu-HMG HCC may be associated with vascular invasion which was suggested to be a poor prognostic factor. These presented results suggested that high fluorescence intensity of gGlu-HMRG associated with an increased risk for early postoperative disease recurrence. The present study is the first one that suggests the utility of gGlu-HMRG-based fluorescence imaging for predicting tumor invasiveness and postoperative disease recurrence, although this will need to be evaluated in additional studies with larger numbers of patients.

In conclusion, fluorescence imaging of tumors after topical administration of gGlu-HMRG enabled rapid and highly specific identification of hepatic malignancies in fresh human specimens. This technique had potential advantages over fluorescence imaging using ICG in that it directly identified cancerous tissues of hepatic adenocarcinoma irrespective of biliary congestion around the tumor. In addition, high fluorescence intensity of gGlu-HMRG associated with increased risks of tumor invasiveness and early postoperative disease recurrence. In the future, fluorescence imaging following direct administration of gGlu-HMRG to the patient’s abdominal cavity may provide surgeons with more accurate information on the extent of cancer spread and on the postoperative behavior of hepatic tumors.

## Methods

### Patients and collection of hepatic specimens

This study was conducted with the approval of the Institutional Ethics Review Board of the University of Tokyo Hospital and was registered in the UMIN Clinical Trials Registry (registration number: UMIN000003655; http://www.umin.ac.jp/ctr/index.htm). Written informed consent was obtained from all patients. The authors confirm that all methods were performed in accordance with the relevant guidelines and regulations.

The study subjects were 95 patients who had undergone liver resection for HCC (48 patients), ICC (8 patients), or CRLM (39 patients) at the University of Tokyo Hospital from July 2010 to February 2014. Simultaneous resection of primary colorectal cancer was performed in 9 of the 39 patients with CRLM.

All patients underwent preoperative ICG retention rate at 15 min (ICGR15) testing. ICG (Diagnogreen, Daiichi Sankyo, Tokyo, Japan) was administered intravenously at a dose of 0.5 mg/kg body weight, and a blood sample was drawn from a different site 15 min later. The test was performed basically 2 days before surgery to estimate hepatic functional reserve^31^. Results are expressed as the percentage of ICG retained after injection (normal value of ICGR15, <10%).

Following liver resection, the surgical specimens were cut in the operating room to obtain the maximal cut surface area of the tumor. The fresh samples were obtained from cut surface area as 3-mm-thicken-slice section containing both cancerous tissue and non-cancerous hepatic parenchyma in the operating room. Then we transferred the fresh samples to the experimental laboratory and imaged within 10 min of resection.

### Fluorescence imaging of resected specimens using gGlu-HMRG

The pharmacological properties and chemical structure of gGlu-HMRG have been described elsewhere (Supplementary Fig. S3)^23^. The fresh hepatic cancer samples were placed on a plate and sprayed with a solution of gGlu-HMRG (50 µM). Fluorescence images of the tissue samples were obtained using a Maestro *In Vivo* Fluorescence Imaging System (CRI, Woburn, MA, USA) with the blue filter setting (excitation 445–490 nm, emission 515 nm, long pass). Fluorescence images were also obtained after spraying paired samples with gGlu-HMRG (50 µM) with or without GGsTop (50 µM), a specific irreversible inhibitor of GGT^32^, under the same conditions.

The fluorescence intensities (FIs) of cancerous tissues and background liver parenchyma were calculated with the Maestro Imaging System by setting regions of interest on the cut surfaces of the tumors or on non-cancerous liver parenchyma at least 3 mm from the cancerous tissues. To minimize the effects of autofluorescence, the signal intensities before gGlu-HMRG administration were subtracted from the FIs of cancerous tissues and background liver tissues at 5 min and 30 min after gGlu-HMRG spraying.

### Fluorescence imaging of tumors after ICG injection

Fluorescence imaging of ICG in the hepatic tumors was also performed with the Maestro Imaging System using the near-infrared filter setting (excitation 710–750 nm, emission 810 nm, long pass). ICG administered during preoperative liver function testing accumulates in differentiated HCC tissues and in non-cancerous hepatic parenchyma with biliary congestion, both enabling visualization of hepatic cancers from the hepatic surfaces^1, 3^. On the cut surfaces of resected specimens, the ICG fluorescence patterns were classified as “cancerous-type” (fluorescence in the cancerous tissue) and “rim-type” (fluorescence in the non-cancerous hepatic tissue surrounding the tumors, but not in the tumors themselves), according to previous studies^1^. The FIs of cancerous tissues and background liver parenchyma without biliary congestion (more than 5 mm apart from the tumor borders) were calculated with the Maestro Imaging System.

### Pathological diagnosis and immunohistochemical staining of tumors

Resected specimens were fixed with formalin and embedded in paraffin for hematoxylin–eosin and immunohistochemical staining of GGT. For immunohistochemistry, the paraffin block was sectioned into 4-µm-thick slices and stained with a mouse monoclonal antibody against a partial recombinant GGT (H00002678-M01; Abnova, Taipei, Taiwan; 1:150 dilution). Immunohistochemical staining was performed according to standard techniques using a Ventana Benchmark XT autostainer (Ventana Medicalc System Inc., Tucson, AZ, USA). Results of the microscopic examinations were evaluated by experienced pathologists (A.U., J.S., and M.F.).

### Statistical analysis

Continuous data are expressed as the median (range). Quantitative and categorized variables were compared using Wilcoxon’s rank-sum test and Fisher’s exact test, respectively. The optimal cut-off FI value at 30 min after gGlu-HMRG spraying for HCC was identified by construction of ROC curves of cancerous tissue and non-cancerous liver parenchyma. The survival curves were constructed using the Kaplan–Meier method and differences were compared using the log-rank test. *P* values < 0.05 were considered statistically significant. Statistical analysis was performed using JMP software (version 9.0.0; SAS Institute Inc., Cary, NC, USA).

## Electronic supplementary material

Supplemental material
