# Supplementary material for: Intraoperative imaging of hepatic cancers using γ-glutamyltranspeptidase-specific fluorophore enabling real-time identification and estimation of recurrence
Source: Sci Rep. 2017 Jun 14;7:3542. doi: 10.1038/s41598-017-03760-3 (PMC5471246; doi:10.1038/s41598-017-03760-3)
Supplement: Supplementary file 1 — Supplemental material [file 41598_2017_3760_MOESM1_ESM.pdf]

Supplementary materials

Title

Intraoperative imaging of hepatic cancers using γ-glutamyltranspeptidase-specific fluorophore enabling real-time identification and estimation of recurrence

Author list

Yoichi Miyata

Takeaki Ishizawa*

Mako Kamiya

Suguru Yamashita

Kiyoshi Hasegawa

Aya Ushiku

Junji Shibahara

Masashi Fukayama

Yasuteru Urano*

Norihiro Kokudo

Supplementary Table S1 Patient clinicopathological characteristics

Characteristics	HCC<br>48 patients	ICC<br>8 patients	CRLM<br>39 patients
Age (years)	68 (34–87)[a]	73 (58–88)	69 (44–84)
Male	41 (85%)	3 (38%)	25 (64%)
Hepatitis B and/or C positive	36 (75%)	2 (25%)	4 (10%)
Preoperative chemotherapy	15 (31%)	1 (13%)	15 (38%)
Liver cirrhosis	19 (40%)	0 (0%)	0 (0%)
Preoperative blood examinations			
GGT (U/L)	48 (16–593)	37 (15–646)	47 (13–436)
Albumin (g/dL)	3.8 (2.6–4.7)	4.3 (3.7–4.8)	3.8 (2.1–4.5)
ICGR15 (%)	11.8 (5.20–41.2)	8.5 (4.50–21.7)	8.1 (4.00–20.7)

[a]Data are the median (range) or *n* (%). HCC, hepatocellular carcinoma; ICC, intrahepatic cholangiocarcinoma; CRLM, colorectal carcinoma liver metastasis; GGT, γ-glutamyltranspeptidase; ICGR15, indocyanine green retention rate at 15 min.

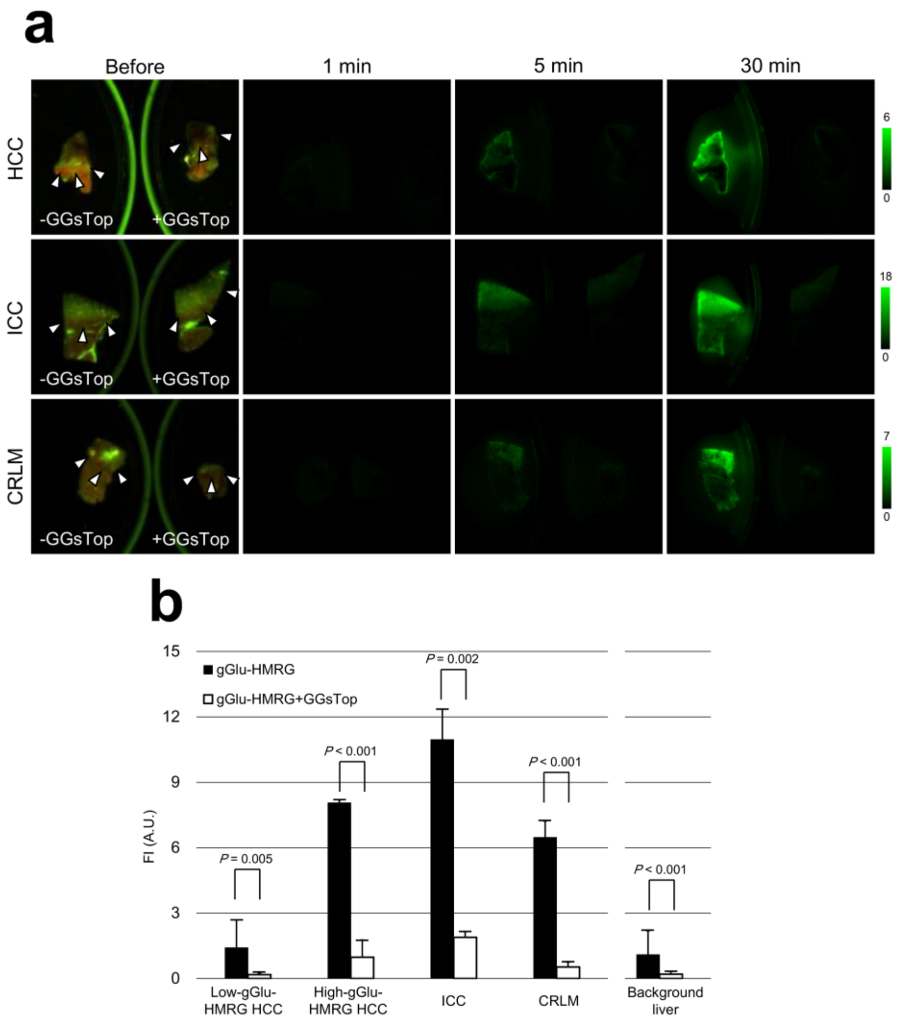

Supplementary Figure S2. Reduction of gGlu-HMRG fluorescence in HCC, ICC, and CRLM by co-application of a GGT inhibitor.

(**a**) Fluorescence images of paired resected specimens before and at 1, 5, and 30 min after topical administration of either gGlu-HMRG alone (left side) or gGlu-HMRG mixed with the GGT inhibitor GGsTop (right side). Fluorescence signals were grossly unidentifiable in the presence of the GGT inhibitor. (**b**) Fluorescence intensities (FIs, in arbitrary units) at 30 min after administration of gGlu-HMRG with or without GGsTop. Data are the median and upper quartile pairs of *n* = 12, 9, 4, 13, and 38 for Low-gGlu-HMRG HCC, High-gGlu-HMRG HCC,

ICC, CRLM, and background liver parenchyma, respectively. Inhibition of GGT significantly reduced the FIs for each tissue type examined

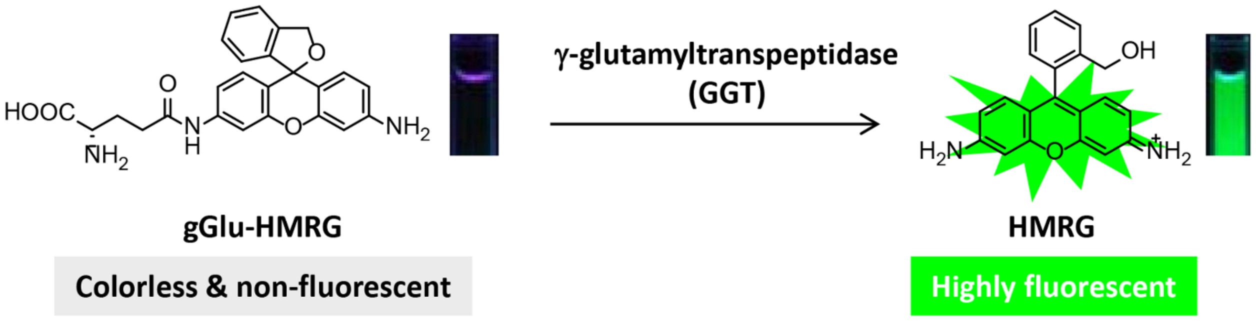

Supplementary Figure S3. Chemical structure of gGlu-HMRG.

gGlu-HMRG was colorless and non-fluorescent fluid in its native state. After administration of GGT, gGlu-HMRG is rapidly hydrolyzed and emit fluorescence with a peak wavelength of 520nm.
